# Epidemiological and genetic variation analysis of emerging porcine circovirus type 2 in Henan Province, 2023

**DOI:** 10.3389/fvets.2025.1598383

**Published:** 2025-04-22

**Authors:** Chaoliang Leng, Jiajing Song, Jiabao Wang, Hongyue Zhai, Camilo Ayra-Pardo, Jiajia Cao, Junjie Li, Yingying Zhao, Hongfei Shi, Dandan Li, Yunchao Kan, Lunguang Yao, Zhijun Tian

**Affiliations:** ^1^Henan Provincial Engineering and Technology Center of Animal Disease Diagnosis and Integrated Control, Henan Key Laboratory of Insect Biology in Funiu Mountain, Henan Province Engineering Research Center of Insect Bioreactor, China-UK International Joint Laboratory for Insect Biology of Henan Province, Nanyang Normal University, Nanyang, China; ^2^Laboratory of Animal Parasitology and Pathology, CIIMAR/CIMAR LA, Interdisciplinary Centre of Marine and Environmental Research, University of Porto, Matosinhos, Portugal; ^3^State Key Laboratory for Animal Disease Control and Prevention, Harbin Veterinary Research Institute, Chinese Academy of Agricultural Sciences, Harbin, China

**Keywords:** porcine circovirus type 2, PCV2d, phylogenetic analysis, genetic variation, new sub-genotype

## Abstract

Porcine circovirus type 2 (PCV2) is a highly adaptable pathogen with significant implications for global swine health. In 2023, we investigated the prevalence and genetic variation of PCV2 in Henan Province, China, by analyzing blood and tissue samples from 380 pigs exhibiting clinical symptoms of PCV2 infection, including reproductive disorders and respiratory diseases. PCR analysis was used to detect PCV2, and viral sequences from 13 positive samples were characterized through phylogenetic and mutational analyses. PCV2 was detected in 56.58% (215/380) of samples. Nucleotide homology among newly identified PCV2 strains ranged from 95.14 to 100%, and 91.18–99.89% compared to 36 global reference strains. Phylogenetic analysis of the ORF2 gene encoding the viral capsid protein Cap identified PCV2a, PCV2b, and PCV2d subtypes, with most sequences clustering into three PCV2d subgroups (PCV2d-1, PCV2d-2, and PCV2d-3). Notably, the PCV2a strain HN230707 exhibited significant genetic divergence, forming an independent branch. Mutational analysis of the Cap protein revealed key amino acid substitutions in conformational epitope regions (T60S, R63T, N77D, V80L, L185M, A191K, and I200T), potentially contributing to immune evasion. Additionally, unique mutations in the nuclear localization signal and conformational epitope regions were identified in PCV2d subgroups. The emergence of genetically diverse PCV2 strains, particularly novel PCV2d sub-genotypes, raises concerns regarding their potential to evade vaccine-induced immunity. These findings highlight the importance of continuous molecular surveillance and the need for updated vaccine strategies to mitigate the impact of PCV2 on global swine health.

## 1 Introduction

Porcine circoviruses (PCVs), belonging to the family *Circoviridae* and genus *Circovirus*, are the smallest known viruses infecting swine. They possess non-enveloped, icosahedral virions measuring 14-17 nm in diameter and contain a covalently closed, single-stranded circular DNA genome of approximately 1.7 kb (1, 2). Four PCV types have been identified (PCV1-PCV4) (2–5). Among these, PCV2 is the primary causative agent of porcine circovirus-associated diseases (PCVDs), a spectrum of conditions that include postweaning multisystemic wasting syndrome (PMWS), porcine dermatitis and nephropathy syndrome (PDNS), respiratory and intestinal disorders, and reproductive failure. These conditions pose a significant threat to global swine production due to their impact on pig health, growth, and mortality (1).

The PCV2 genome contains two major open reading frames (ORFs): ORF1 and ORF2 (6, 7). ORF1 encodes the Rep protein, a 35.7 kDa replicase essential for viral replication (8). ORF2 encodes the 27.8 kDa Cap protein, the sole component of the viral capsid and the primary target of host immune responses (9–11). The Cap protein displays functional variability across its amino acid (aa) sequence. In addition to the nuclear localization signal (NLS, aa1-41), Cap contains several antigenic structural domains, including genotype-specific domains (aa86-91, aa190, aa191, aa206, and aa210) and conformational epitopes (aa47-85, aa165-200, and aa230-233) (12, 13). These epitopes, particularly those recognized by neutralizing monoclonal antibodies (MAbs) at aa145-162, aa175-192, and aa231-233, play a key role in immune evasion. Mutations at residues such as aa59, aa86, aa88, aa91, aa151, aa190, aa191, and aa206 influence the immune response to PCV2 MAbs (14, 15), while mutations at aa59, aa60, aa190/151 and aa131/191 significantly alter the virus antigenicity and neutralization potential. Based on ORF2 sequence variations, at least nine genotypes (PCV2a to PCV2i) have been identified to date (16–23). Additionally, ORF3 protein induces apoptotic responses and contributes to viral pathogenesis *in vitro* and *in vivo* (24–27). ORF4 protein inhibits caspase activity and suppresses the proliferation of CD4 + and CD8 + T cells, further modulating the immune response (28).

Since its emergence, PCV2 has undergone two major genotype shifts, from PCV2a to PCV2b in 2003 and subsequently to PCV2d in 2012, which now dominates globally (29, 30). Despite widespread vaccination, PCV2 continues to evolve, raising concerns about vaccine efficacy and the emergence of novel strains capable of evading immune protection. Henan Province, one of the largest pig farming regions in China, has experienced fluctuating PCV2 prevalence in recent years. While infection rates declined from 2017 to 2022, a significant resurgence was observed in 2023, particularly in some large-scale farms (31). The objective of this study was to investigate the prevalence and genetic diversity of PCV2 in Henan Province. To this end, we conducted PCR analysis on blood and tissue samples from 380 pigs that exhibited clinical symptoms such as piglet wasting, growth retardation, respiratory signs (e.g., coughing and fever), and reproductive issues in sows (e.g., mass abortion and stillbirth) from different farms across the province. Whole-genome amplification was then performed on 13 positive samples, and the genomic sequences were analyzed for homology with reference strains from GenBank. The comparative analysis focused on the Cap protein sequences, revealing key genetic variations and differences between the new strains and the reference strains. These findings provide valuable insights into the genetic variability of PCV2 and its implications for vaccine development and disease management.

## 2 Materials and methods

### 2.1 Sample collection, DNA extraction, and PCR analysis

**Figure 1:**
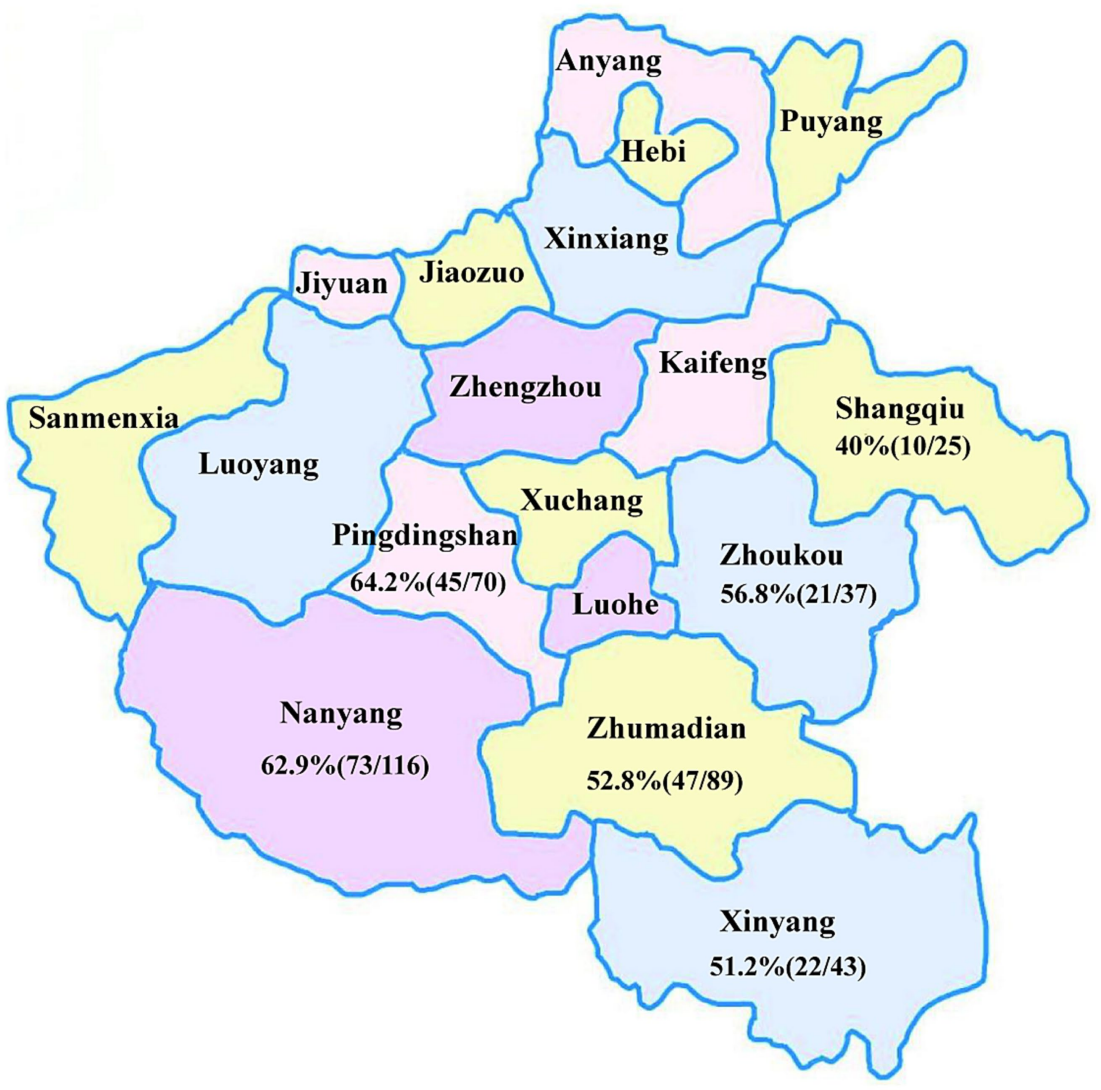
Regional distribution of samples and PCV2 positivity rates in Henan Province.

A total of 380 samples, including blood, lungs, and spleens, were collected from pigs exhibiting clinical signs of reproductive disorders and respiratory diseases. These samples were obtained from farms located in six cities within Henan Province (Nanyang, Zhumadian, Pingdingshan, Xinyang, Zhoukou, and Shangqiu) in 2023 (Figure 1). All samples were stored at −80°C until further processing. Viral genomic DNA was extracted from 200 μL serum or tissue homogenate using a Simply P Virus DNA/RNA Extraction kit (Hangzhou BORI Technology, Hangzhou, China) following the manufacturer’s instructions. The extracted DNA was stored at −80°C until use.

PCR amplification was performed using standard PCV2-specific primers (31). The PCR protocol consisted of an initial denaturation step at 95°C for 5 min, followed by 34 cycles of denaturation at 95°C for 30s, annealing at 55°C for 30s, and extension at 72°C for 30s, with a final elongation step at 72°C for 10 min. The PCR products were purified and recovered using the E.Z.N.A Gel Extraction kit (Guangzhou Feiyang Biological Engineering, Guangzhou, China) and subsequently cloned into the pMD18-T vector. Positive clones were identified by PCR and sent to Sangon Bioengineering (Shanghai, China) for Sanger sequencing. The positive rates of PCV2 were determined based on the combined results of PCR amplification and sequencing analysis.

### 2.2 Whole genome amplification and sequencing of PCV2

Viral genomic DNA was extracted from thirteen PCV2-positive samples by using a Simply P Virus DNA/RNA Extraction kit (Hangzhou BORI Technology, Hangzhou, China) and used as the template for PCR amplification. The complete PCV2 genomes were amplified in overlapping segments using a set of PCV2-specific primer pairs designed to span the entire viral genome (31). The PCR-amplified products were purified and subsequently ligated into the pMD18-T T/A cloning vector (Takara Bio, Seoul, Korea). The ligation products were then transformed into *Escherichia coli* TOP10 competent cells (Thermo Fisher Scientific, Hanover Park, IL, United States). Positive colonies were screened by colony PCR and sent to Sangon Bioengineering (Shanghai) for Sanger sequencing. The sequencing data were assembled and analyzed using SeqMan in the DNASTAR software package (version 7.10, DNASTAR, Madison, Wisconsin, United States) to generate complete viral genome sequences.

### 2.3 Identification and phylogenetic analysis

To analyze the genomic nucleotide (nt) sequences and ORF2 nt sequences of the 13 newly obtained PCV2 strains and elucidate their phylogenetic relationships, the assembled sequences were aligned with the genomic sequences of 36 representative PCV2 strains from different sub-genotypes retrieved from GenBank. Whole-genome and ORF2 sequence similarity analyses were conducted for the 13 newly identified strains and the 36 reference strains using the MegAlign program within the DNASTAR software package. A phylogenetic tree was constructed based on the genome sequences of the 13 new strains and the 36 reference sequences. The ORF2 sequences, commonly used to monitor viral genetic variation, were used to classify PCV2 into distinct genotypes based on their sequence variability. According to the MODELS program in the Molecular Evolutionary Genetics Analysis (MEGA) software (v.11.0), the ORF2 phylogenetic tree was constructed using the Maximum Likelihood method with the Hasegawa-Kishino-Yano model and a discrete Gamma distribution (HKY + G) to account for evolutionary rate differences among sites (32). Bootstrap values were calculated using 1,000 replicates.

### 2.4 Analysis of amino acid variability in the cap protein

The Cap protein of PCV2 contains multiple antigenic structural domains, including key amino acid (aa) sites that are critical for the recognition and binding of the host’s neutralizing antibodies to the virus (12, 33). To identify mutations and assess sequence variability, we performed a comparative analysis of the aa sequences of the Cap protein from 13 newly obtained PCV2 strains and 36 reference strains. Multiple sequence alignments were performed using the ClustalW method in the MegAlign program (DNASTAR package, version 7.10).

## 3 Results

### 3.1 Positive rate and regional distribution

PCR was performed on 380 suspected PCV2-positive samples collected from various cities of Henan Province. The results revealed an overall positive rate of 56.58% (215/380). Regional positivity rate varied, with the highest rate observed in Pingdingshan (64.2%, 45/70), followed by Nanyang (62.9%, 73/116), Zhoukou (56.8%, 21/37), Zhumadian (52.8%, 47/89), Xinyang (51.2%, 22/43) and Shangqiu (40%, 10/25) (Figure 1). Notably, the positivity rates in Pingdingshan, Nanyang and Zhoukou exceeded the overall positivity rate.

### 3.2 Sequence identity analysis

**Figure 2:**
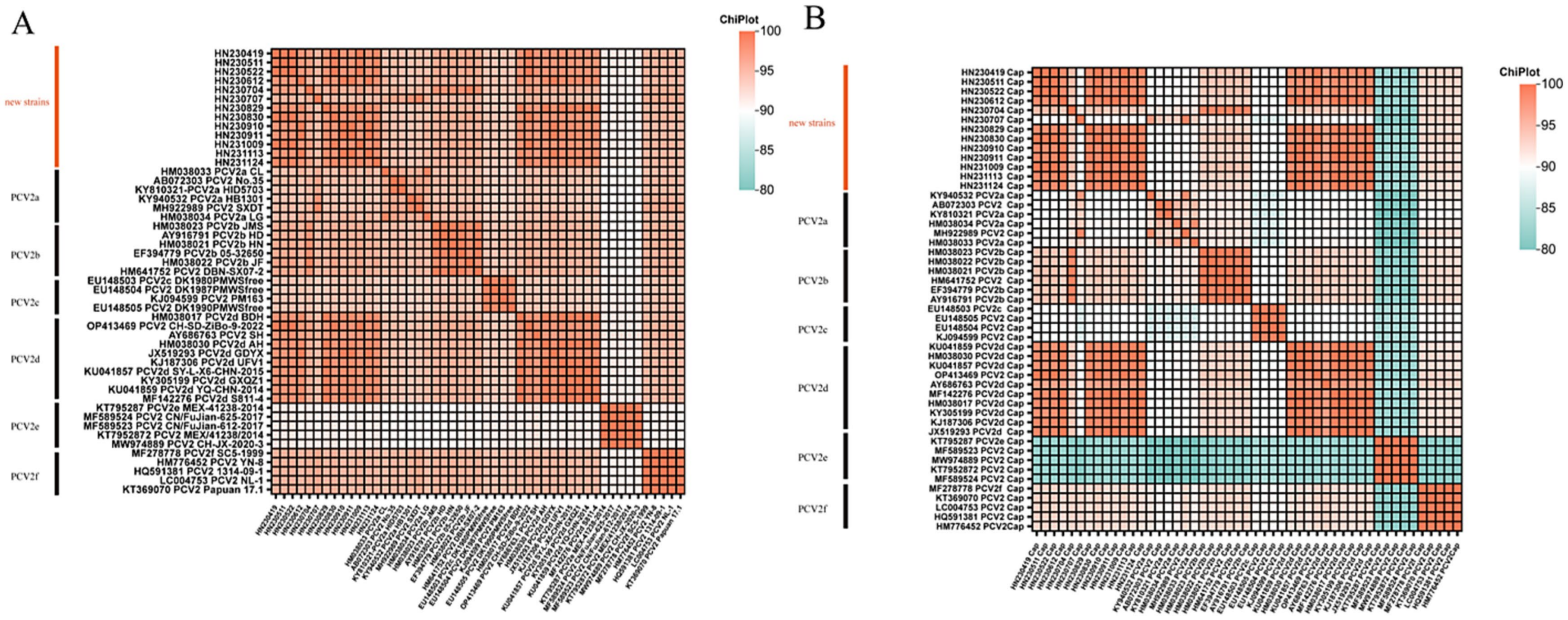
Heatmap illustrating the percentage identity between 13 new PCV2 isolates and 36 reference strains based on whole-genome sequences **(A)** and ORF2 gene sequences **(B)**.

A total of 13 PCV2-positive samples were amplified by PCR, sequenced, and assembled to obtain whole genome sequences, all of which were 1,767 nt in length. Pairwise comparisons showed that the whole genome sequences of the 13 PCV2 endemic strains shared 95.19–100% sequence identity among themselves, while their identity with the 36 PCV2 reference genome sequences from GenBank ranged from 91.23% to 99.89% (Figure 2A). Specifically, the sequence identity between the new strains and the vaccine strains PCV2a LG (HM038034), PCV2b DBN-SX07-2 (HM641752), PCV2d SH (AY686763), and the Danish representative strain PCV2c DK1980PMWSfree (EU148503) ranged from 95.08%–96.55%, 95.81%–98.47%, 96.21%–98.42%, and 94.12%–95.02%, respectively (Supplementary Table 1).

Analysis of the ORF2 sequences showed that the new PCV2 strains shared 89.93–100% sequence identity among themselves. Their identity with the vaccine strains PCV2a LG (HM038034), PCV2b DBN-SX07-2 (HM641752), PCV2c DK1980PMWSfree (EU148503) and PCV2d SH (AY686763) ranged from 90.50%–94.03%, 92.60%–99.29%, 86.81%–90.21%, and 91.21%–97.45%, respectively (Supplementary Table 1). Pairwise comparisons of ORF2 sequences between the new strains and reference strains revealed sequence identities ranging from 80.75% to 100% (Figure 2B).

Further analysis indicated that the new strain HN230707 exhibited the highest sequence identity (96.38%–99.10%) with reference strains of the PCV2a genotype. Similarly, the new strain HN230704 showed the highest identity (98.13%–99.43%) with reference strains of the PCV2b genotype. The remaining 11 new strains displayed the highest identity (95.14%–99.89%) with reference strains of the PCV2d genotype. Based on these findings, the 13 new strains were preliminarily classified as belonging to the PCV2a, PCV2b, and PCV2d genotypes. Notably, the new strain HN230707 exhibited greater genetic variability, with sequence identities of only 94.12%–96.55% compared to four reference strains. These results suggest that the PCV2d genotype is currently the predominant strain circulating in Henan Province, and a significantly divergent new strain emerged in 2023.

### 3.3 Phylogenetic analysis

**Figure 3:**
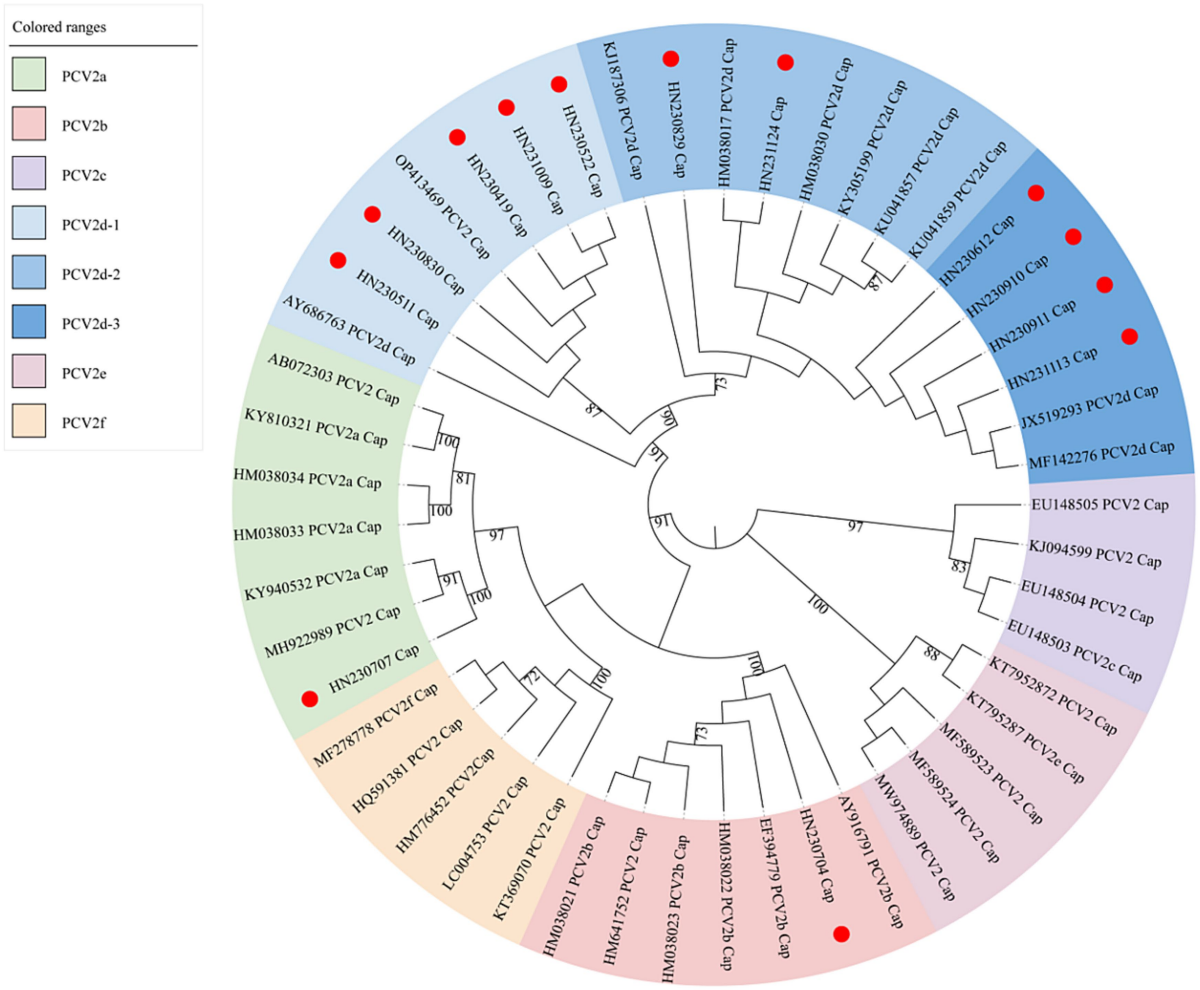
Maximum-likelihood phylogenetic tree constructed from ORF2 gene sequences of 13 new PCV2 isolates and 36 reference sequences. New PCV2 strains identified in this study are indicated with red circles.

To investigate the genetic diversity of PCV2 in Henan Province in 2023, a phylogenetic tree was constructed using the Cap protein sequences of 13 new strains and reference strains. As shown in Figure 3, the new PCV2 strains were classified into three genotypes: PCV2a, PCV2b and PCV2d.

Among the new strains, HN230707 clustered with the PCV2a reference strains, confirming its classification as a PCV2a genotype. Similarly, HN230704 clustered with the PCV2b reference strains, indicating it belongs to the PCV2b genotype. The remaining 11 new strains formed a larger cluster with the PCV2d reference strains. Within this cluster, five strains (HN230419, HN230511, HN230522, HN230830, HN231009) grouped into a distinct sub-cluster with the reference strains PCV2 CH-SD-ZiBo-9-2022-CAP (OP413469) and PCV2d SH (AY686763). A second sub-cluster included the new strains HN230829 and HN231124, which grouped with reference strains PCV2d UFV1 (KJ187306), PCV2d BDH (HM038017), PCV2d AH (HM038030), PCV2d GXQZ1 (KY305199), PCV2d SY-L-X6-CHN-2015 (KU041857), and PCV2d YQ-CHN-2014 (KU041859). A third sub-cluster comprises reference strains PCV2d GDYX (JX519293) and PCV2d S811-4 (MF142276), which grouped with four new strains: HN230612, HN230910, HN230911, and HN231113. These three sub-clusters were designated as PCV2d-1, PCV2d-2, and PCV2d-3, respectively.

Interestingly, the new strain HN230829, which emerged in 2023, formed a relatively independent branch within the PCV2d-2 sub-genotype, suggesting significant genetic divergence. Similarly, HN230707 clustered with the PCV2a reference strain but was distantly related to them, indicating potential genetic variability. These results suggest that the PCV2d genotype remains the predominant strain circulating in Henan Province, while PCV2 continues to evolve, leading to increased genetic diversity and complex epidemiological patterns.

### 3.4 Amino acid sequence analysis of the cap protein

**Figure 4:**
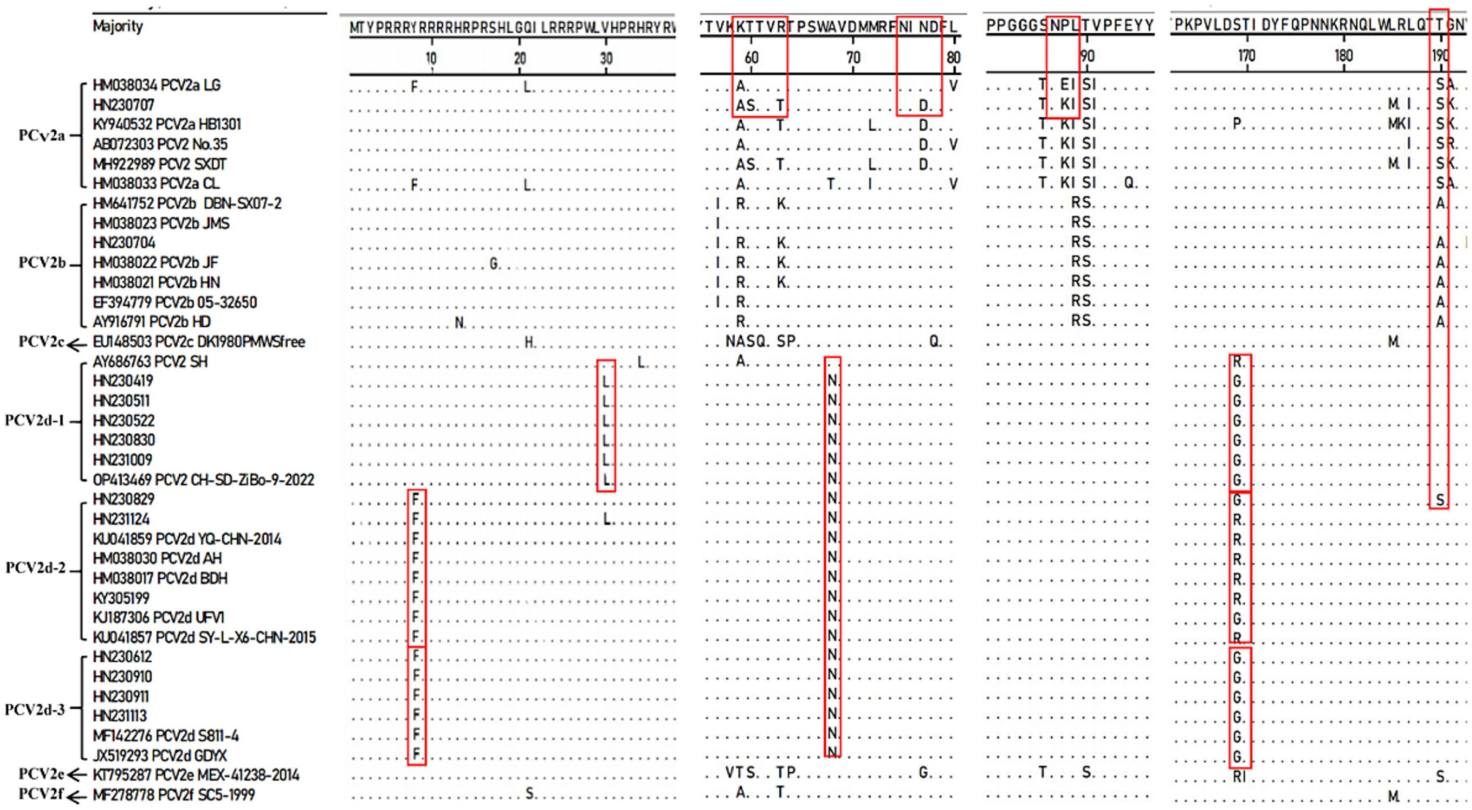
Amino acid sequence analysis of the Cap protein comparing new PCV2 isolates with reference strains.

To further characterize the molecular features of the newly identified PCV2 strains, we compared the aa sequences of their Cap proteins with those of reference strains using MegAlign software (Figure 4). The results revealed consistent aa mutations across different PCV2 subtypes, which facilitate the identification of the new strains.

Compared to the PCV2a LG strain (HM038034), the PCV2d strains exhibited a consistent A68T mutation within the conformational epitope region aa 47–85. The newly identified PCV2d-1 sub-genotype displayed two additional mutations: V30L in the NLS region and S169G/R within the conformational epitope region aa 165–200, with V30L being unique to this sub-genotype. Similarly, PCV2d-2 and PCV2d-3 strains shared the Y8F mutation in the NLS region and S169G/R mutation within the conformational epitope region aa 165–200. However, at aa169 of the Cap protein, most PCV2d-2 strains carried an R, while all PCV2d-3 strains carried a G. These consistent aa mutations in the Cap protein sequences of PCV2d-1, PCV2d-2, and PCV2d-3 further support the existence of multiple sub-genotypes within PCV2d.

In addition, the newly identified strain HN230707 exhibited four mutations (T60S, R63T, N77D, and V80L) within the conformational epitope region aa 47–85 and three mutations (L185M, A191K, and I200T) within the conformational epitope region aa 165–200 when compared to the PCV2a LG (HM038034) strain. Furthermore, within the genotype-specific structural domains aa 86–91, the new strain HN230707 harbored an E88K mutation compared to PCV2a reference strains. Interestingly, all analyzed PCV2d strains contained a unique A68N mutation within the conformational epitope region aa 47–85, distinguishing them from other PCV2 strains. In contrast to other PCV2d strains, the PCV2d strain HN230829 exhibited a T190S mutation within the conformational epitope region aa 165–200, which was identical to that of PCV2a strains.

Overall, these results indicate that the Cap protein of some newly identified PCV2 strains carries more aa mutations compared to the reference strains, particularly the vaccine strain PCV2a LG (HM038034). Such genetic variations may contribute to immune evasion, potentially reducing the efficacy of current vaccines.

## 4 Discussion

In this study, we investigated the prevalence and genetic diversity of PCV2 in Henan Province, China, in 2023. Our findings reveal a sharp increase in PCV2 positivity rates in large-scale pig farms since late 2022, accompanied by significant genetic variability in circulating strains. PCV2 remains one of the most prevalent pathogens responsible for PCVD, posing a serious threat to swine health and leading to substantial economic losses in the global swine industry (34, 35). Although widespread vaccination has helped control PCV2 to some extent, the genetic diversity of the virus enables persistent infections in large-scale pig farms, negatively impacting economic efficiency and the overall development of the pig industry (36). Henan, a major pig farming province in China, has its swine industry concentrated in Nanyang, Zhumadian, Pingdingshan, Xinyang, Zhoukou and Shangqiu. Since late 2022, the PCV2 positivity rate has increased in some large-scale pig farms, causing a range of economic repercussions (31). To assess the prevalence of PCV2 in Henan Province in 2023, we collected samples from these areas and performed PCR on 380 suspected cases of infection. The results showed a PCV2 positive rate of 56.58% (215/380), with regional rates ranging from 40 to 64.2%, indicating widespread PCV2 infection in Henan. Previous studies have reported positivity rate exceeding 40% across different regions of China, with variations likely influenced by statistical time- and geographic-based factors (37).

Our recent research showed a declining PCV2 positivity rate in Henan from 2017 to 2022, decreasing from 58.65% to 7.87% (31). However, since late 2022, the detection rate has surged reaching 56.58% in 2023, similar to 2017 levels (31). This resurgence may be linked to the low price of pigs in China since 2022, prompting farms to modify immunization programs to reduce costs. This likely resulted in decreased PCV2 vaccine administration and overall herd immunity, facilitating the rapid spread of the virus. Additionally, the concurrent widespread prevalence of porcine reproductive and respiratory syndrome virus (PRRSV), PCV3, and PCV4 in recent years has likely exacerbated PCV2 infections (38).

The Cap protein, encoded by the ORF2 gene, is the sole structural protein of PCV2, and serves as a key marker for monitoring genetic variation and genotyping (11, 39). Previous studies indicate that the dominant PCV2 genotype in China has shifted twice, with PCV2d emerging as the predominant genotype in 2012 (40, 41). To elucidate the genotypic distribution of PCV2 in Henan in 2023, we selected 13 samples from the 215 positive cases, which exhibited typical clinical symptoms of PCV2 infection and tested negative for other major swine viruses. Complete genome sequences were obtained through sequence assembly using the SeqMan program. Phylogenetic analysis of the ORF2 gene classified these strains into PCV2a (7.69%), PCV2b (7.69%), and PCV2d (84.62%) subtypes, confirming the continued predominance of PCV2d subtype in Henan. This finding aligns with the broader trend in China, where PCV2d has largely replaced PCV2b as the dominant genotype since 2012.

The phylogenetic analysis also identified two independent clades, represented by PCV2b strain HN230704 of and PCV2a strain HN230707. Among the 11 isolated PCV2d strains, three distinct subclades—PCV2d-1, PCV2d-2, and PCV2d-3—were identified, marking the first detailed sub-genotypic classification of PCV2d. This classification enhances our understanding of PCV2d genetic diversity, facilitating more precise epidemiological tracking and the development of regional disease-control strategies and vaccines. Notably, the emergence of the new sub-genotypes PCV2d-1 and PCV2d-3 in large numbers over a short period of time suggests that genetic variation among PCV2d strains may be more frequent and rapid than previously thought. Continuous monitoring of PCV2d genotypic variation is, therefore, crucial, both for epidemiological assessment and the design of next-generation vaccines. However, whether these sub-genotypes will become dominant in the future is uncertain and requires further surveillance.

The PCV2a strain HN230707 was isolated from a farm where sows, despite routine PCV2 vaccination, experienced severe reproductive failures, including abortions, stillbirths, and mummified fetuses. Testing conducted by our laboratory ruled out other major reproductive pathogens, leading to the hypothesis that genetic mutations in this strain may have enhanced its pathogenicity or enabled it to evade vaccine-induced immunity. Current commercial PCV2 vaccines, primarily based on PCV2a subtype, have demonstrated cross-protection against PCV2b and PCV2d under laboratory conditions (42). However, the recent surge in PCV2 positivity, the emergence of the HN230707 variant, and the prevalence of PCV2d-1 and PCV2d-3 sub-genotypes strongly suggest that existing vaccines may not provide comprehensive protection under real-world conditions (42–44). This underscores the urgent need for the development of updated vaccines tailored to evolving PCV2 strains.

Given the high variability of the Cap protein, we analyzed the aa sequence of the Cap protein from newly isolated strains of the predominant PCV2d. The results confirmed that all 11 new strains showed specific aa mutations within the conformational epitope region aa 47–85, consistent with PCV2d classification. Additionally, PCV2d-1, PCV2d-2 and PCV2d-3 showed distinct aa mutations within the conformational epitope region aa 165–200 and the NLS region aa1-41, reinforcing the presence of multiple sub-genotypes within PCV2d. The new strains HN230707 and HN230829 showed unique aa mutations in the conformational epitope region aa 175–192, a critical region for recognition by PCV2 neutralizing Mab, suggesting that these mutations may affect viral neutralization (45). Moreover, HN230707 harbored multiple aa mutations within the conformational epitope regions aa 47–85 and aa 165–200, which could alter its antigenicity, pathogenicity, and vaccine-induced immune protection, potentially contributing to reproductive failures in affected farms. These findings emphasize the necessity of continuous PCV2 genomic surveillance to track viral evolution and its impact on vaccine efficacy, enabling the timely development of disease prevention and control strategies.

## 5 Conclusion

In summary, our study revealed a sharp increase in PCV2 positivity rates in some large-scale pig farms in Henan Province since late 2022, accompanied by significant genetic variability in circulating strains. These developments warrant increased attention from China’s pig industry and emphasize the need for enhanced surveillance of PCV2 epidemiology and genetic diversity. Strengthening monitoring efforts will provide critical insights for the scientific prevention and control of PCV2, ensuring better management of its economic and health impacts on swine production.

## Data Availability

The datasets presented in this study can be found in online repositories. The names of the repository/repositories and accession number(s) can be found in the article/Supplementary material.
