# Supplementary material for: Epidemiological and genetic variation analysis of emerging porcine circovirus type 2 in Henan Province, 2023
Source: Front Vet Sci. 2025 Apr 22;12:1598383. doi: 10.3389/fvets.2025.1598383 (PMC12053293; doi:10.3389/fvets.2025.1598383)
Supplement: Supplementary file 1 [file Table_1.docx]

Supplementary Material

**Epidemiological and genetic variation analysis of** **emerging porcine circovirus type 2 in Henan Province, 2023**

Chaoliang Leng^1^^*^, Jiajing Song^1^, Jiabao Wang^1^, Hongyue Zhai^1^, Camilo Ayra-Pardo^2^, Jiajia Cao^1^, Junjie Li^1^, Yingying Zhao^1^, Hongfei Shi^1^, Dandan Li^1^, Yunchao Kan^1^, Lunguang Yao^1^, Zhijun Tian^3*^

**Correspondence:** Corresponding Author: [lenghan1223@126.com](mailto:lenghan1223@126.com); [tianzhijun@caas.cn](mailto:tianzhijun@caas.cn)

**Supplementary Table 1.** Sequence identity analysis between 13 new PCV2 isolates and 4 representative strains

| New PCV2 strains | Accession nos. | PCV2a LG (HM038034) | PCV2b DBN-SX07-2 (HM641752) | PCV2c DK1980PMWS free (EU148503) | PCV2d SH (AY686763) |
| --- | --- | --- | --- | --- | --- |
| HN230704 | PQ661744 | 95.59^a^/92.88^b^ | 98.47/99.29 | 95.02/90.21 | 97.00/94.04 |
| HN230707 | PQ661745 | 96.55/94.03 | 95.81/92.60 | 94.12/86.81 | 96.21/91.21 |
| HN230419 | PQ661740 | 95.14/90.64 | 96.72/94.75 | 94.85/89.93 | 98.13/97.02 |
| HN230511 | PQ661741 | 95.08/90.64 | 96.66/94.75 | 94.74/89.79 | 98.08/96.74 |
| HN230522 | PQ661742 | 95.14/90.64 | 96.72/94.75 | 94.85/89.93 | 98.25/97.02 |
| HN230830 | PQ661747 | 95.14/90.50 | 96.55/94.61 | 94.96/90.07 | 98.08/96.88 |
| HN231009 | PQ661750 | 95.14/90.64 | 96.77/94.75 | 94.79/89.93 | 98.08/97.02 |
| HN230829 | PQ661746 | 95.48/90.92 | 96.04/93.76 | 94.34/89.22 | 97.62/96.88 |
| HN231124 | PQ661752 | 95.31/91.06 | 96.21/93.62 | 94.45/89.36 | 98.42/97.30 |
| HN230612 | PQ661743 | 96.21/90.92 | 96.32/93.76 | 94.68/89.22 | 97.34/97.03 |
| HN230910 | PQ661748 | 96.15/90.92 | 96.21/93.76 | 94.74/89.22 | 97.45/97.30 |
| HN230911 | PQ661749 | 96.21/91.06 | 96.26/93.90 | 94.79/89.36 | 97.51/97.45 |
| HN231113 | PQ661751 | 96.21/91.06 | 96.26/93.90 | 94.79/89.36 | 97.51/97.45 |

a: PCV2 whole genome

b: ORF2 gene
